# Isoflurane causes muscle contraction in *Drosophila melanogaster* despite inducing hyperpolarized state

**DOI:** 10.17912/micropub.biology.001732

**Published:** 2026-03-14

**Authors:** Joshua Griffith, Jiwoo Kim, Robin Cooper

**Affiliations:** 1 College of Medicine, University of Kentucky, Lexington, Kentucky, United States; 2 Biology, University of Kentucky, Lexington, Kentucky, United States; 3 The Gatton Academy of Mathematics and Science, Bowling Green, Kentucky, United States; 4 Model Laboratory School, Richmond, Kentucky, United States

## Abstract

Isoflurane is a commonly used volatile anesthetic; however, its mechanisms are not well characterized. To better understand mechanisms of action, isoflurane was applied to model larval *Drosophila* muscle preparations. Membrane potential, evoked transmissions, and observations of muscle behavior were recorded. Muscle contraction was not inhibited by dantrolene (10 mM) or ryanodine (100 µM). Thapsigargin (1 mM), which depleted the sarcoplasmic reticulum of Ca ^2+^ , inhibited muscle contraction. Blebbistatin blocked contractions induced by isoflurane, allowing for membrane hyperpolarization. Isoflurane releases stored intracellular calcium from the sarcoplasmic reticulum of the skeletal muscle, causing muscle contraction despite direct hyperpolarization of the membrane.

**Figure 1. The effect of isoflurane on larval Drosophila muscle:**
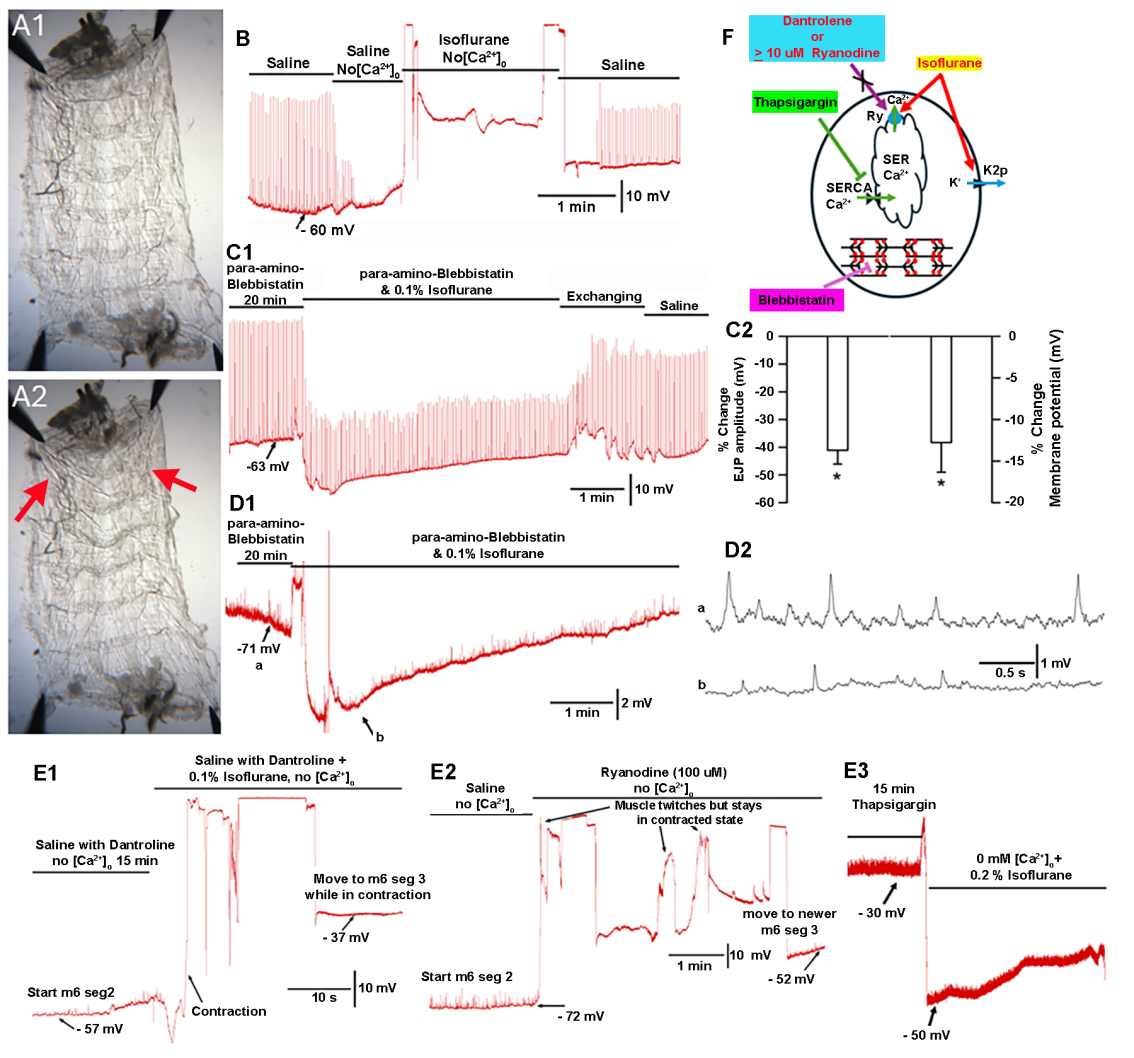
**(A1)** In saline without Ca ^2+^ , muscle fibers are relaxed. **(A2)** In saline without Ca ^2+^ and with isoflurane (0.1%), pronounced muscle contraction occurs (arrows). **(B)** Evoked excitatory junction potentials (EJPs) recording in muscle 6 (m6) stopped occurring in saline without Ca ^2+^ and upon exposure to isoflurane (0.1%) in saline without Ca ^2+^ muscle contraction occurring, making it difficult to maintain an intracellular membrane potential due to muscle contraction bending the inserted microelectrode and losing the recording. Removing the bent microelectrode out of the fiber and re-recording from the same fiber in a contracted state after exchanging the saline to one with Ca ^2+^ revives the EJPs with the muscle remaining in a contracted state. **(C1)** Pre-incubation for 20 minutes with para-amino-Blebbistatin (100mM) in saline with Ca ^2+^ (1 mM) and then recording EJPs, at 0.5 Hz stimulation frequency, and exposure to para-amino-Blebbistatin with 0.1% isoflurane in saline with Ca ^2+^ resulted in hyperpolarization of the membrane potential while producing EJPs although reduced in amplitude. Flushing the bath with fresh saline revived the membrane potential, as well as the EJPs. **(C2)** The amplitude of the evoked EJPs before and after exposure to 0.1% isoflurane while incubated in para-amino-Blebbistatin for 6 preparations. The mean (+/-SEM) percent difference for the 6 preparations is shown. &nbsp; **(D1)** Pre-incubation for 20 minutes with para-amino-Blebbistatin (100 µM) in saline with Ca ^2+^ (1 mM), followed by exposure to para-amino-Blebbistatin with 0.1% isoflurane in saline with Ca ^2+^ , resulted in membrane hyperpolarization while still producing miniature EJPs (mEJPs). **(D2)** The amplitude of the miniature EJPs (mEJPs; spontaneous quantal responses) before (2 min period) and after (2 min period) exposure to 0.1% isoflurane while incubated in para-amino-Blebbistatin for 6 preparations. **(D2)** &nbsp;The amplitudes of the mEJPs decreased in amplitude to the point of starting to become lost in the base line noise. This occurred for all 6 preparations. The **D2a** enlarged trace to from the trace is shown in **D1** and likewise for **D2b** . &nbsp; **(E1)** Incubation for 15 minutes with dantrolene (10 µM) in saline without Ca ^2+^ and then the same composition of saline but with 0.1% isoflurane resulted in muscle contractions and loss of the intracellular recording. Moving the microelectrode to another contracted muscle fiber (i.e., m6 one segment caudal) still illustrated a depolarized and contracted fiber. &nbsp; **(E2)** Incubation in a saline without Ca ^2+^ and then saline without Ca ^2+^ but with ryanodine (100 µM) resulted in muscle contractions and loss of the intracellular recording. Moving the microelectrode to another contracted muscle fiber (i.e., m6 one segment caudal) still illustrated a depolarized and contracted fiber.&nbsp; **(E3)** Incubation for 15 minutes with thapsigargin (1 mM dissolved in DMSO) in a saline without Ca ^2+^ and then the same composition of saline but with 0.1% isoflurane blocked muscle contraction. The effect of solvent DMSO is controlled for as it was the same concentration during the pre-incubation as during exposure with isoflurane. Thus, the membrane potential recording was maintained and a hyperpolarization induced by isoflurane can be observed. Lowered extracellular Ca ^2+^ reduces the block on the NALCN (Na ^+^ leak channels); thus, membrane potential depolarizes with zero Ca ^2+^ extracellular. All six preparations (p<0.05) showed strong hyperpolarization without prolonged contractions, thus illustrating the action of isoflurane rapidly hyperpolarizing the membrane even when the muscle is not contracting. **(F)** A representative model of a larval body wall muscle indicating pre-incubation of 15 minutes with dantrolene (10 mM) or ryanodine (100 µM) did not block isoflurane’s ability to release Ca ^2+^ from the activating the ryanodine receptor (RY) on the sarcoplasmic reticulum (SER). Exposure to ryanodine (100 µM) resulted in muscle contraction and possibly induced even more Ca ^2+^ release via calcium induced calcium release from the SER. Pre-incubation of 15 minutes with thapsigargin (1 mM) in saline without Ca ^2+^ blocked the contractions induced by 0.1% isoflurane exposure. Pre-incubation of 20 minutes with para-amino-Blebbistatin (100 µM) in saline with Ca ^2+^ and exposure to 0.1% isoflurane in saline containing Ca ^2+^ blocked muscle contraction while hyperpolarizing the membrane potential. In summary, isoflurane appears to release Ca ^2+^ from SER within the muscle fiber, resulting in contraction while simultaneously producing a hyperpolarization of the membrane potential, potentially by activating a subtype of K2P channels.

## Description

Isoflurane is a volatile anesthetic commonly used in hospital settings to induce and maintain general anesthesia in patients undergoing surgery (Miller et al., 2023). Isoflurane is a nonflammable, FDA-approved drug that has been clinically applied since 1979 (Hawkley et al., 2025). It is used in both humans for surgery and animal models for research and is characterized by a strong, pungent odor.

Isoflurane causes dose-dependent effects on various sites of action in the human body, such as ion channels (e.g., GABA receptors) and specific organ systems (e.g., heart; Hawkley et al., 2025). In general, anesthetics selectively affect the nervous system by directly acting on proteins, such as ligand-gated ion channels (Franks and Lieb, 1994). For instance, isoflurane can hyperpolarize the cellular membrane potential by interacting with two-pore domain potassium channels (K2P; Berg-Johnsen and Langmoen, 1990; Felisberti et al., 1997; Maciver et al., 1991; Franks and Lieb, 1988; Patel et al., 1999). Isoflurane can also induce hyperactivity by enhancing sodium leak channels (NALCN; Ou et al., 2019). In addition, isoflurane can trigger malignant hyperthermia, a disorder in calcium regulation in skeletal muscles that is associated with uncontrolled muscle contraction (Ravaei et al., 2020; Gelb, 2014). However, despite ongoing research, the mechanisms of action of isoflurane have not been well characterized (Miller et al., 2023; Osman et al., 2023).

The larval *Drosophila* model provides many advantages for studying the effects of isoflurane, particularly the intracellular mechanisms of skeletal muscle contraction. Specific advantages include having identifiable single muscle fibers with non-spiking (i.e., graded) evoked depolarizations. Sarcomeric contractions of the larval *Drosophila* are similar to that of mammalian hearts due to Ca ^2+^ voltage-gated channels in the plasma membrane as well as release from sarcoplasmic reticulum (SER).

Thus, in this study, we investigated the effects of isoflurane exposure on skeletal muscle contraction using the larval *Drosophila* model (A1, A2, and B). We also assessed the effects of dantrolene, ryanodine, and thapsigargin to determine their ability to inhibit contraction in single muscle fibers. To prevent muscle contraction while recording membrane potential, para-amino-Blebbistatin was tested to examine if it uncouples myosin and actin in larval *Drosophila* body wall muscles to allow intracellular membrane monitoring (C1, C2, D1, and D2).

Pre-incubation for 15 minutes with dantrolene (10 mM) did not block the induced muscle contractions by isoflurane (0.1%) in a saline of zero Ca ^2+^ ( *n* =6). Despite that ryanodine > 100 µM is known to block the RY receptor, it resulted in strong contractions in the larval *Drosophila* muscle model ( *n* =6) in a saline of zero Ca ^2+^ (E1, E2 andE3). Pre-incubation for 15 minutes with thapsigargin (1 mM) in saline with no Ca ^2+^ nearly eliminated muscle contraction induced by isoflurane (0.1%). Intracellular recordings during muscle contractions resulted in membrane damage. Recordings in statically contracted muscles revealed membrane potentials of resting membranes of normally uncontracted states. Pre-incubation of 20 minutes in para-amino-Blebbistatin with saline containing Ca ^2+^ (1 mM) blocked isoflurane-induced muscle contractions. Blocking muscle contraction with para-amino-Blebbistatin induced hyperpolarization of the muscle membrane potential, which was able to be measured, upon exposure to isoflurane. Evoked excitatory junction potentials (EJPs) and miniature EJPs (quantal responses) were slightly depressed during the isoflurane-induced hyperpolarization (mean of 17.6 mV). Flushing with fresh saline revived the membrane potential and amplitudes of the EJPs and mEJPs. A general model of action by isoflurane on the larval muscle is shown in F.

## Methods

**Animals**

*Drosophila melanogaster* (fruit fly) have remained isogenic in the lab since initial acquisition and have been kept at ~20°C and ~75% humidity on a 12-hour light/dark cycle in partially filled vials of cornmeal-agar-dextrose-yeast medium. Canton S *Drosophila melanogaster* larvae in the early third instar were used for all experiments.

**Dissection and Recordings**

The technique of dissecting larvae and measuring membrane potential was described previously (Kim et al., 2025). Larvae were dissected and pinned at four corners with ventral side up. All segmental nerves were transected close to the larval brain. The segmental nerve for individual segments was pulled into a suction electrode to stimulate the motor neurons while monitoring the evoked synaptic responses in muscle 6 (m6) in segment 2 with an intracellular electrode. A sharp intracellular electrode (30 to 40 megaohm resistance) filled with 3M KCl impaled the fiber. An Axoclamp 2B (Molecular Devices, Sunnyvale, CA, USA) amplifier and 1 X LU head stage were used. Data were collected using a PowerLab/4SP (ADInstruments, Colorado Springs, CO, USA), and analyzed with LabChart 7.0 (ADInstruments, Colorado Springs, CO, USA), which were recorded on a computer at a 20 kHz sampling rate along with the use of a NPI GMbH filter (type EPMS07 DPA 2F, from Adam and List Associate, LTD., 1100 Shames Drive, Westbury, NY 11590, USA) at low pass filtered at 3.0 kHz with no high pass filtering.

A modified basal HL3 saline was used (NaCl 70 mM, KCl 5 mM, MgCl2·6H2O 10 mM, NaHCO3 10 mM, Trehalose 5 mM, sucrose 115 mM, BES 25 mM, and CaCl2·2H2O 1 mM, pH 7.1; Stewart et al., 1994; De Castro et al., 2014; Bidros et al., 2024). The pH of the saline was maintained at 7.2. Saline compounds were obtained from Sigma *-* Aldrich, St. Louis, MO, USA.

Isoflurane (Sigma-Aldrich, St. Louis, MO, USA.) was directly added to saline (small volume 5 to 10 ml vials) right before use due to the possibility of isoflurane vaporizing if time before use was prolonged, vortexed on high for 2 minutes, and then directly applied to the dissected preparations. The concentration used: 0.1% (v/v). To prepare thapsigargin, 1 mg was dissolved in 150 µL of DMSO and 1.35 mL of saline without Ca ^2+^ to produce a concentration of 1 mM. When no CaCl _2_ is added to the saline&nbsp; and exposed to the muscle, the resting membrane potential will depolarize due to relieving the block of Ca ^2+^ on the Na ^+^ leak channels (NALCN) (Hana et al., 2026). Para-amino blebbistatin was prepared by dissolving 1 mg in 5 µL of DMSO and adding HL3 saline with Ca ^2+^ to produce a 100 µM solution. Ryanodine was prepared by adding 1mg to 200 µL of 100% EtOH and diluting to 100 µM concentration using HL3 saline with Ca ^2+^ . Dantrolene was prepared by diluting to 10uM concentrations HL3 saline with Ca ^2+^ to produce a concentration of 10 µM. The effect of isoflurane alone is measured when pre-incubated in thapsigargin and DMSO containing saline. The effect of &nbsp;EtOH at 9.87 % v/v is of no concern on ryanodine’s directs effect of inducing muscle contraction, as EtOH at 0.4 M after 15 min at 22 ^o^ C did not produce depolarization (Magazanik and Vyskocil, 1979) and generally EtOH decreases synaptic transmission without causing contractions at the arthropod glutamatergic NMJ (Strawn and Cooper, 2002). Regardless of past reports, we examined &nbsp;the effect of EtOH at 9.87 % for the effect on membrane potential and muscle contraction at the larval NMJ. Within 2 minutes of exposure to saline which was previously 9.87 % v/v EtOH vortex and left open for the duration of the experiments (30 minutes),&nbsp; the membrane potential decreased&nbsp; on average 2 mV for 7 preparation and none of the preparations showed muscle contractions, unlike the case for exposure to ryanodine.

*Observations*

Muscle contractions were visually confirmed.

## Reagents

**Table:** 

NaCl (Sigma-Aldrich, St. Louis, MO, USA.)
KCl (Sigma-Aldrich, St. Louis, MO, USA.)
MgCl2.6H2O (Sigma-Aldrich, St. Louis, MO, USA.)
NaHCO3 (Sigma-Aldrich, St. Louis, MO, USA.)
L-Trehalose (Sigma-Aldrich, St. Louis, MO, USA.)
sucrose (Sigma-Aldrich, St. Louis, MO, USA.)
N,N-bis(2-hydroxyethyl)-2-aminoethanesulfonic acid (Sigma-Aldrich, St. Louis, MO, USA.)
CaCl2.2H2O (Sigma-Aldrich, St. Louis, MO, USA.)
Dantrolene (Sigma-Aldrich, St. Louis, MO, USA.)
Isoflurane (Sigma-Aldrich, St. Louis, MO, USA.)
*Drosophila melanogaster* strains Bloomington Drosophila Stock Center (RRID:BDSC_64349).
Ryanodine (TOCRIS 614 McKinley Place NE Minneapolis, MN 55413 USA)
Thapsigargin (TOCRIS 614 McKinley Place NE Minneapolis, MN 55413 USA)
Para-Amino-Blebbistatin from Cayman Chemical Company 1180 East Ellsworth RoadAnn Arbor, Michigan 48108 USA
